# Supplementary material for: Are carbon emissions trading and green financial instruments synergistic? -Comprehensive quantitative research based on content analysis
Source: PLoS One. 2024 Mar 7;19(3):e0298601. doi: 10.1371/journal.pone.0298601 (PMC10919601; doi:10.1371/journal.pone.0298601)
Supplement: S1 Data — (ZIP) [file pone.0298601.s001.zip › Data/地级市Z_F工作报告.zip!/┴╔─■/┤≤┴1⁄4╩╨/└┤╫╘╣1⁄2╓┌║┼í╛┬φ┐╦╩2╛▌═°í┐_2008.docx]

各位代表：

　　我代表市政府向大会报告工作，请予审议，并请市政协各位委员提出意见。

2007年和过去五年工作回顾

　　2007年，市政府在党中央、国务院，省委、省政府和市委的正确领导下，以邓小平理论和“三个代表”重要思想为指导，深入贯彻落实科学发展观，带领全市人民锐意进取，开拓创新，埋头苦干，圆满完成了全年和本届政府的各项任务。

　　预计全市地区生产总值突破3000亿元，增长17%以上；万元GDP综合能耗下降4%，主要污染物排放总量降低3.4%；完成全社会固定资产投资1900亿元，增长30%；完成地方财政一般预算收入267.8亿元，增长36.5%；实现社会消费品零售总额984亿元，增长17.2%；完成外贸进出口总额355亿美元，增长21%；实际使用外资31.6亿美元，增长41%；城镇居民人均可支配收入15108元，增长13.2%；农村居民人均纯收入8369元，增长19.8%；城镇登记失业率降低到2.46%。全市经济社会发展步入又好又快的轨道。

　　1、保税港区正式封关运作，国际航运中心辐射功能大幅提升。大窑湾保税港区建设快速推进，一期通过国家验收并投入运行，在全国试行快速通关操作模式，政策和功能优势开始显现，已有45家企业落户。完成港口基础设施投资70亿元，新增生产泊位12个，增加吞吐能力2240万吨。长兴岛公共港区3个7万吨级通用泊位竣工并通航，大窑湾集装箱码头二、三期等大型专业化泊位建设加快推进。金窑铁路复线全线通车，大连铁路集装箱中心站开工建设。大窑湾疏港高速公路建成通车，沈大与丹大高速公路连接线主体完工，长兴岛疏港高速公路和滨海公路开工建设。预计全年完成海港货物吞吐量2.2亿吨，增长11.2%；集装箱吞吐量381.3万标准箱，增长18.7%；空港旅客吞吐量726万人次，增长14.3%。大连口岸辐射作用进一步增强，国际航运中心建设实现了历史性突破。

　　2、“两区一带”建设快速启动，先进制造业聚集效应初步形成。以发展先进装备制造业为重点，集中布局了一大批具有较强支撑作用的产业项目。大窑湾临港区域汽车配套产业初具规模，德国大众和一汽集团汽车核心部件生产基地已经形成。大连湾临海区域形成了海洋工程、船用曲轴、核电和风电设备等重型装备产业集群。渤海岸线STX造船、万邦修造船项目开工建设，中远造船项目前期工作全面启动。大孤山半岛对二甲苯、对苯二甲酸等石化深加工项目加快推进。国家战略石油储备基地一期主体工程完成，中石油保税库一期投产。以集成电路为主的微电子产业发展取得重大突破，一期投资25亿美元的英特尔超大规模集成电路项目开工建设，拉开了我市建设亚洲重要集成电路产业基地的序幕。全市共实施工业项目2458个，总投资1837亿元。实现规模以上工业增加值1100亿元，增长27%；利税增长70%。实现高新技术产业增加值575亿元，增长36.9%。重大项目的建设为我市未来发展打下了坚实基础。

　　3、现代服务业发展势头强劲，支撑作用日趋显现。制定现代服务业发展规划，确定了重点推进的四大产业、八大领域。现代物流业快速发展，普洛斯、凯雷、万邦等跨国公司投资的物流项目相继启动。金融业对全市经济贡献率大幅提高，金融机构利润超过100亿元，增长70%。大连银行获准跨区域经营并在天津市设立分行，美国花旗银行等7家金融机构落户大连。大连商品交易所实现由单一农产品期货向综合性期货市场转型，期货交易额达到12万亿元，增长1倍。软件产业持续高速发展，实现销售收入215亿元，增长48.3%；出口7.2亿美元，增长60%。旅顺南路软件产业带建设全线展开，腾飞软件园一期投入使用，东软国际软件园即将竣工，大连天地软件园动工建设，龙头软件园开始启动，旅顺大学园正式开园。在国际数据公司全球交付指数城市排名中，大连位列中国第一、世界第五名。旅游观光业取得突破，我市获得“中国最佳旅游城市”称号。老虎滩海洋公园•老虎滩极地馆被批准为国家首批5A级旅游景区，莱茵海岸、老虎滩渔人码头开工建设，大连海滨国家地质公园开园。举办展会92个，平均规模增长46%。现代服务业发展对全市经济拉动作用明显增强。

　　4、新农村建设扎实推进，县域经济快速发展。市财政向涉农区市县投入资金35亿元，各项支农惠农政策全面落实，农业产业结构调整和农村改革不断深化，县域经济的自主增长能力显著增强。涉农区市县预计实现地区生产总值1520亿元，增长19.8%；完成财政收入74.7亿元，增长38.4%。农业获得全面丰收，粮食总产量突破30亿斤，创历史最高水平。农林牧渔及服务业总产值增长19%。农业产业化水平明显提高。新建、扩建农产品加工龙头企业63家，发展农民专业合作组织212家，有70万农户进入农业产业化体系。农产品质量安全管理和动物检疫体系进一步健全。农村基础设施和生态环境建设不断加强。新修公路460公里，新建沼气工程4069个，建成各类水源工程1161项。完成590户农民生态移民搬迁。植树1亿株，森林覆盖率达41.5%，基本建成森林防火和病虫害防治体系。54个新农村示范村的基础设施建设和村屯环境整治进展顺利，5个乡镇生活污水和垃圾综合治理示范工程开工建设。广大农村的面貌发生了可喜变化。

　　5、招商引资取得重大突破，对外开放成果丰硕。积极整合开放资源、创新开放举措，对外开放质量和水平全面提高。坚持招商选资，新批投资总额5000万美元以上的外资项目39个，引进世界500强投资项目11个，一批牵动力大、具有战略意义的外资项目落户大连。使用外资结构进一步优化，在引进外资总量中，优势产业项目占76%。先导区和工业园区的产业集聚效应显现，三个先导区实际使用外资占全市的60%；“一岛十区”新批外资项目33个，实际使用外资4.7亿美元。全市引进内资突破300亿元。对外贸易增长方式明显转变，机电产品、高新技术产品出口大幅提高。外贸出口突破190亿美元，增长21.4%。企业“走出去”步伐加快，新批境外企业22家。成功承办了中国夏季达沃斯年会，充分展示了大连城市魅力，90多个国家和地区的6000多位政界、商界领袖和各界贵宾汇聚我市，为大连更好地融入世界、加快国际化进程创造了良好条件。大连被誉为具有良好国际形象的新领军城市。

　　6、改革创新进程加快，经济发展活力显著增强。国有大中型企业改革任务基本完成，历史遗留问题基本得到解决，国有企业实现利润增长80%。华锐铸钢上市已获证监会批准。国有资产监管体系进一步完善。理顺了市对县财政管理体制，县区级财政部门预算改革有效推进。税收属地化管理体制已经形成。

　　民营经济不断壮大，发展环境明显改善。万达、亿达、华信等8家企业成为世界经济论坛成长型企业签约会员。中小企业融资担保体系初步形成，民营担保公司发展到55家，担保额超过90亿元，建成市级小企业创业基地11个、中小企业公共服务平台12个，新创小企业突破1万家。傅氏国际和绿诺科技在美国成功上市。民营经济对全市经济贡献率提高到57.2%。

　　自主创新能力得到增强。组织实施了光电子等126项自主创新重大专项，获国家、省级科技奖励65项，发明专利申请增长20%，我市荣获国家知识产权示范城市创建市称号。创新孵化体系建设稳步推进，中科院大连科技创新园建设进展顺利。新增孵化面积100万平方米，孵化企业1000多家。企业创新体系建设加快，新认定国家级和省级企业技术中心9家。重工•起重集团自主研发的大型船用曲轴正式下线，兆瓦级风电设备生产规模列中国第一位，大连机床集团、光洋科技、大森数控研发的中高档数控系统和机床关键功能部件在全国领先，一重大连加氢公司百万千瓦核电压力容器项目达到国际先进水平。全年开发新产品1680余项，投产1000项。自主创新在经济发展中显现出巨大的驱动力量。

　　7、基础设施建设力度加大，城市环境进一步优化。实施城建重点项目180项，完成投资315亿元。发展城市公共交通，建成首条快速公交线路（BRT），快轨三号线续建工程主体完工，完成201路有轨电车线路改造工程。城市地铁、海底隧道项目前期工作启动。煤气新厂二期、香海热电厂二期竣工投产。庄河电厂一期提前半年并网发电，红沿河核电站主体工程开工建设。国家节水型社会建设示范市通过验收。实施了长海县大长山岛引水工程。节能减排及环境建设取得显著成效，各项指标居全国前列。星海湾水源热泵一期工程投入运行，小平岛水源热泵、北海热电厂再生水利用工程加快建设，我市成为全国唯一的海水源热泵技术示范城市。砬夏河整治全线竣工，春柳河、马栏河二期等污水处理厂试运行。采取有力措施，彻底清理南部海域养殖物，海洋环境明显改善。拆除水泥立窑58座、旋窑7座，提前完成540万吨落后水泥产能淘汰任务。拆除中心城区锅炉186台。东北特钢大连基地、大化集团等企业整体搬迁进程加快。夏家河海滨公园、明珠公园开园，西郊国家森林公园建设顺利推进，城区绿化覆盖率达到43.3%。我市“蓝天白云”的良好环境得到国内外的广泛赞誉。

　　8、社会事业加快发展，文明城市建设取得新成果。坚持把教育放在优先发展地位。全市农村义务教育公用经费全部纳入市县两级财政预算。投入3000万元，完成135所办学设施标准化学校建设。建成6个职业教育实训基地，职业教育设施进一步完善。大医、大外新校区正式启用，学校面貌和教学条件发生了历史性变化。大连职业技术学院进入国家高职高专“211工程”。中国大连高级经理学院成为国家唯一培训国有大型企业高管人员基地。文化事业健康发展。创作生产了一批集思想性、艺术性和观赏性为一体的文化艺术作品，电视剧《守望幸福》荣获“五个一”工程奖。社区文化广泛开展，专业文艺团体送戏下乡演出100场，为农民放映电影1.2万场，城乡群众文化生活进一步活跃。卫生事业发展加快。完成了大医一院三部、传染病、皮肤病、结核病4所医院建设，启动了市中心医院扩建工程。市县两级财政投资1.8亿元，为区市县卫生院、妇幼保健院配备医疗仪器设备。我市被确定为全国健康城市首批试点城市。群众体育、竞技体育取得新成绩，市体育中心开工建设。计划生育率99.69%，人口自然增长率0.92‰，低生育水平保持稳定。

　　精神文明和社会建设取得新成果。广泛开展向全国道德模范方永刚、全国职业道德标兵王亮等学习教育实践活动。我市荣获第六轮全国双拥模范城称号。加大投入、强化监管，深入开展安全隐患排查和专项整治，各类事故得到有效遏制。受理群众来信来访下降18.2%，信访秩序明显好转。深入实施公安“三基”工程建设，进一步完善社会治安防控体系，严厉打击各种犯罪，社会治安保持稳定。“四五”普法、人民调解工作和平安大连建设取得明显成效。

　　新闻出版、档案、民族、宗教、人防、侨务、对台、妇女儿童、残疾人、老龄、民兵和预备役工作均取得较好成绩。

　　我市经济社会统筹协调发展，和谐大连建设取得令人瞩目的成就。

　　9、民生工程全面实施，人民群众生活水平持续改善。采取一系列重大举措，加大各级财政投入，民生质量明显提高。实施积极的就业政策，累计筹集再就业资金6.2亿元，发放小额担保贷款1亿元，实现就业16.1万人，其中创业就业1.9万人、大龄困难人员就业9591人，有就业愿望的零就业家庭人员全部实现就业。投资1.3亿元建设高校毕业生就业服务中心，建成高校毕业生就业见习基地102家。启动了“困难家庭毕业生就业援助工程”，对登记失业和困难家庭毕业生进行100%的就业援助。

　　社会保障水平稳步提高，社会保险覆盖范围不断扩大，征缴社会保险费97.1亿元，征缴率达97.6%。建立城市低收入家庭专项救助制度，进一步提高失业保险和最低工资标准。投入低保救助资金2.1亿元，15万城乡低保对象得到保障。完善了城市困难家庭、农民工子女就学制度和扶困助学体系，农村27万名中小学生和城区1万多名贫困学生享受到“两免”政策，困难家庭子女就学问题得到解决。努力解决群众“看病难、看病贵”问题，实施城市困难居民基本医疗救助制度，实现了市内四区城镇居民基本医疗保险全覆盖。出台了农村困难居民医疗救助办法。新农合筹资标准提高到80元，参合农民达211万人，参合率94.7%。新建农村区域性中心敬老院16所，新增城乡养老床位2527张。大幅提高百岁老人生活补贴标准，新建残疾人康复站307个，启动市孤残儿童康复中心建设。

　　建立健全了以住房公积金、廉租住房、经济适用住房为重点的住房保障体系。为市内四区1469户“双特困”家庭发放了廉租住房租赁补贴。归集住房公积金51.2亿元，向2.2万名职工发放住房公积金贷款36.5亿元。开工建设经济适用住房85万平方米，为低收入家庭提供住房1万余套。完成了凌水湾、香周路等10个区域的旧城区拆迁。帮助农村特困居民改造危房1500户。

　　启动“食品安全城市”建设，开展了产品质量和食品安全专项整治工作，深入整顿和规范食品市场秩序，加大打假治劣力度，保障了食品安全。

　　市政府年初确定的为民办18件实事和重点民生工程全部完成。

　　10、行政效能大幅提高，服务型政府建设成效显著。全面开展“行政效能年”建设。整合、界定政府工作部门内部的行政许可职能，实现了部门内部行政审批集中办理。整合成立了市政府行政投诉中心。创新行政效能监督手段和方式。在10个政府部门进行了引入ISO9000质量管理体系试点，规范了工作流程，提高了行政效率。加强四级公共服务体系建设，形成了覆盖全市的行政服务、便民服务网络。开辟了重点项目审批服务“绿色通道”。优化行政审批流程，总体审批时限比上年缩短了53%。重点推进政务信息公开、办事公开，提高了公共服务的透明度。企业与政府打交道的时间缩短为年均14.6天，处于全国先进水平。审计监督工作加强，规范了财政资金管理和使用。进一步完善应急管理体制和应急预案体系，夯实了应急管理的基层和基础工作。全面开展公务员素质培训，与新加坡建立公务员培训长期合作关系，各类培训收效明显，公务员队伍整体素质得到提升。各级政府行政效能的提高，得到市民和企业的充分肯定。

　　市政府及政府各部门自觉接受人大及其常委会法律监督和工作监督，认真执行人大决议，坚持重大事项向人大常委会报告制度。自觉接受政协的民主监督，实行重大决策向政协通报，积极采纳各民主党派、工商联、无党派人士参政议政意见。1621件市人大代表建议和政协提案全部办结，满意率达95%以上。加强与工会、共青团、妇联等群团组织的联系，认真听取和采纳人民群众对政府工作的意见和建议。

　　在肯定成绩的同时，我们也清醒地看到，一些制约全市经济社会发展的困难和问题依然存在。一是经济结构仍不尽合理，产业优化升级、企业自主创新、节能减排任务还很艰巨。二是环境容量和土地等资源的有限性对经济社会发展的制约不断增加。三是城镇就业和再就业压力仍然较大，市民收入水平相对较低，部分消费品价格水平上涨幅度较大，低收入群众生活还比较困难。四是城市局部交通拥堵问题亟待解决。这些问题必须在今后工作中认真加以解决。

　　各位代表，本届政府任期即将结束。五年来，我们牢记使命，不负重托，遵循科学发展观的要求和全市人民的意愿，努力解放思想，开拓创新，紧紧抓住东北老工业基地振兴、进一步扩大沿海开放和辽宁“五点一线”沿海经济带建设等重大历史机遇，锲而不舍攻坚克难，励精图治加快发展，抢占了对外开放的制高点，形成了率先全面振兴的良好态势，保持了在区域竞争中的优势地位。今日的大连正在成为世人瞩目、蓬勃向上、富有活力的国际化城市。

　　过去的五年，是综合经济实力大幅跃升、老工业基地振兴取得重大突破的五年。我们规划了“一个中心、四个基地”的宏伟蓝图，始终抓住经济结构战略调整这一主线，深化改革，扩大开放，推进自主创新，加快改造传统产业，大力发展先进制造业、高新技术产业和现代服务业，布局和实施了一批重大产业项目，提升了产业能级和国际竞争力。五年来，经济增长持续加速，经济效益不断提高，经济后劲明显增强。2007年地区生产总值是2002年的2.1倍，财政收入是2002年的2.7倍，固定资产投资是2002年的5.2倍。我市综合经济实力跃上一个新的台阶。

　　过去的五年，是城市综合服务功能全面增强、国际化进程明显加快的五年。我们坚持国际化标准，不断加强城市基础设施建设和环境改造，进一步提高城市的承载力和综合服务功能，为加快国际航运中心和东北亚重要国际城市建设提供了坚实支撑。累计实施城建项目768项，总投资1026亿元，是上一个五年的5.6倍，城市功能、生态环境明显改善。完成港口建设投资295亿元，增加泊位56个，2007年港口吞吐能力是五年前的3倍，集装箱吞吐量是五年前的2.8倍。累计实际使用外资130亿美元，是上一个五年的2.3倍。先后承办了亚欧经济部长会议、WTO小型部长会议、中国夏季达沃斯年会等重要国际会议，城市的国际知名度和影响力大大提高。

　　过去的五年，是城乡一体化进程加快、县域经济发展突飞猛进的五年。我们坚持区域统筹协调发展，把“三农”工作摆在更加重要的位置，采取有力措施支持县域经济快速发展。市财政累计向涉农区市县转移支付资金140多亿元。优化产业和重大项目布局，在区市县形成了以“一岛十区”为重点、具有较强承载力的产业基地。五年来，涉农区市县地区生产总值年均增长17.1%，地方财政一般预算收入年均增长22.8%，社会主义新农村建设取得显著成效。

　　过去的五年，是以民生为重点的社会建设全面展开、城乡居民得到实惠最多的五年。我们坚持统筹经济与社会协调发展，加快推进各项社会事业发展和社会管理进步，创造更多更好的条件满足广大市民日益增长的物质和文化需求，切实改善民生质量。城乡教育协调、均衡发展，公共卫生体系加快建设，城乡文化全面繁荣，体育工作取得成效，各项社会事业实现了历史性进步。市县两级财政直接用于民生方面的支出362亿元，就业规模日益扩大，社会保障体系进一步完善，城乡居民收入明显提高，城镇居民人均可支配收入连续5年、农民人均纯收入连续4年保持两位数增长，困难群众的生产生活条件显著改善，广大人民群众生活水平全面提高。

　　过去的五年，是政府自身建设和管理创新不断加强、政府行政效能显著提高的五年。我们坚持牢记宗旨、执政为民，不断加强政府自身建设，切实转变职能、转变作风、转变工作方式，实现管理创新，着力提高行政效率，努力建设为民务实清廉的政府。先后开展了“软环境建设年”、“行政效能年”建设活动，认真落实建设服务型政府的各项措施，为经济社会快速发展提供有力保障。市级行政许可项目累计削减和调整了82%，年检项目累计削减了53.8%，政府机关工作作风明显好转，政府行政效率大大提高。

　　各位代表，过去的五年，我们取得了令人瞩目的成就。这一切都得益于党中央、国务院，省委、省政府和市委的正确领导；得益于历届政府打下的坚实基础；得益于市人大、市政协和各民主党派、工商联、人民团体、无党派人士对政府工作的有效监督和支持。中、省直单位和外地驻连机构给予了我们多方面的支持和帮助。驻连部队和武警广大官兵心系第二故乡，积极支持和投身地方建设。国内外各界朋友情系大连，为我市经济社会发展做出了重要贡献。广大市民积极参加经济和社会建设，在城市动迁等涉及切身利益的重大问题面前，在抗击非典疫情、战胜“3•4风暴潮”、创建全国文明城市和中国最佳旅游城市、筹办夏季达沃斯年会等重大活动中，顾全大局、甘于奉献，给予了充分的理解和支持。广大进城务工人员远离家乡，为大连的建设做出了积极贡献。还应指出的是，本次大会后，一些政府领导同志因为各种原因将离开工作岗位，他们在届内尽职尽责，勤奋努力，为大连的发展和政府建设做了大量卓有成效的工作。正是这些来自于以上各方面的关心和支持，使全市形成了团结奋斗、开拓进取的大好局面，大连才有美好辉煌的今天。对此，我代表市政府表示衷心的感谢！

今后五年目标任务和2008年主要工作

　　各位代表，过去五年的工作，为大连今后的发展积蓄了强大的后劲。国际产业的加速转移，为大连扩大开放、更好地参与国际经济循环提供了难得机遇。老工业基地振兴战略的深入实施，为大连加快发展创造了有利条件。环渤海和东北两大经济区的快速崛起，使大连的城市功能优势更加突出并将大有作为。

　　今后五年工作的指导思想是：认真贯彻党的十七大精神，高举中国特色社会主义伟大旗帜，以邓小平理论和“三个代表”重要思想为指导，深入贯彻落实科学发展观，加快转变经济发展方式，完善社会主义市场经济体制，大力推进改革开放和自主创新，着力优化经济结构和提高经济增长质量，切实加强节能减排和生态环境保护，扎实推进国际航运中心、新型产业基地和社会主义新农村建设，更加注重改善民生和促进社会和谐，推动经济社会又好又快发展，率先实现老工业基地全面振兴。

　　主要发展目标是：实现地区生产总值年均增长14%以上，力争2012年比2007年翻一番；万元GDP综合能耗年均降低4%，主要污染物排放总量降低10%；全社会固定资产投资年均增长16%；地方财政一般预算收入年均增长16%；社会消费品零售总额年均增长15%；实际使用外资年均增长10%；城镇居民人均可支配收入和农村居民人均纯收入年均增长12%。今后五年工作要重点把握好以下几个问题：

　　——坚持好字优先、好中求快，加快转变经济发展方式。持续提升经济增长的质量和效益，加快推进经济发展向质量型转变。把握世界经济发展趋势和规律，优先发展先进制造业和现代服务业，加快推进产业结构向高级化转型，把大连建设成为全球软件和服务外包新领军城市、亚洲重要的集成电路产业基地、具有国际竞争力的装备制造业基地和东北亚现代服务业聚集中心。加快形成节约能源资源和保护生态环境的产业结构、增长方式、消费模式，营造可持续发展的生态环境，走生产发展、生活富裕、生态良好的文明发展之路。

　　——发挥龙头作用，开创率先实现全面振兴和对外开放的新局面。认真落实温家宝总理在大连视察时的重要讲话要求，充分发挥龙头作用，加快发展，优化服务，为推动东北振兴和对外开放做出更大的贡献。努力放大保税港区政策和功能优势，加强区域合作，带动东北对外开放。切实提高使用外资质量，围绕促进产业结构升级和引进先进技术，加大招商引资力度，完善优势产业链条，壮大优势产业集群。加快开放先导区、“一岛十区”体制创新和发展步伐，使之尽快成为全市开放型经济发展的核心功能区，走在辽宁沿海经济带开发开放的前列，创造参与全球化和国际竞争的新优势。

　　——加大改革攻坚力度，完善推进科学发展、促进社会和谐的体制机制。全面推进综合配套改革，进一步解决好经济社会发展中的深层次矛盾和问题。加大国有资本调整和国有企业重组力度，深化国有企业公司制股份制改革，推动符合条件的企业尽快上市。继续优化促进非公有制经济发展的制度环境，引导非公有制经济健康发展。推进财税、金融、投融资体制改革，完善要素市场体系，健全社会信用体系，充分发挥市场配置资源的基础性作用。深化行政管理体制改革，建设廉洁高效政府。

　　——加快建设创新型城市，增强城市核心竞争力。坚持创新驱动，加快形成以科技创新为基础的新的竞争优势。强化企业在创新中的主体地位，推进以企业为主体、市场为导向、产学研紧密结合的创新平台建设。大力推进开放式创新，按照优势互补、互利共赢的原则，加快引进消化吸收再创新，充分利用全球科技资源，增强自主创新能力，培育面向全球化、以科技和人才资源为支撑的创新型产业和企业。牢固树立人才是第一资源的理念，建立健全人才工作体系，构建人才高地，以更多的人力资源替代物质资源。充分挖掘各类教育资源，创新人才培养模式，优化人才结构，加快形成人才与产业发展的互动机制。

　　——树立现代规划理念，构建全域发展新格局。加快实施城市规划修编，按照“全域谋划、城乡统筹、沿海牵动、两翼推进”的思路，依托黄、渤海沿线的产业园区，继续推进产业的全域布局，加快功能性重大基础设施建设，形成以城市带动农村、以沿海带动全域、以工业化带动城市化的“V”字形发展新格局。对土地资源进行认真规划，科学划分功能区，优化土地开发格局。把生态环境建设放在突出位置，以我市所拥有的资源禀赋和城市承载力为基础，确定城市发展的合理容量和潜力空间。加强森林公园和湿地公园建设，形成“城林交融、路连林隔”的绿色城市空间结构，营造良好的生态宜居环境。严格实施滨海岸线规划，推进城市向海滨发展，让城市靠近岸线，打造以城区为主体、以黄海和渤海沿岸为两翼的大城市格局。

　　——更加注重改善民生，共建共享和谐大连。要把改善民生作为今后五年政府工作的重要任务，进一步加大财政投入，更好地解决人民群众最关心、最直接、最现实的利益问题，努力实现学有所教、劳有所得、病有所医、老有所养、住有所居，推进和谐大连建设。办好人民群众满意的社会事业，推进教育、文化、卫生、体育等事业不断迈上新的台阶，努力为市民提供更多优良的公共服务产品。持之以恒地实施好各项民生工程，大力推进就业和再就业，积极发展创业式就业；建立和完善住房保障体系，全面解决低收入群众的住房困难；增加居民收入，提高居民收入在国民收入分配中的比重；完善覆盖城乡的社会保障体系，确保困难群体的基本权益得到有效保障。全面加强社会管理，营造安定和谐的生活环境。

　　各位代表，新一届政府今后五年的任务十分繁重，我们要在市委的正确领导下，深入贯彻落实科学发展观，带领全市人民艰苦奋斗，努力把大连建设成为一个经济发达、环境优美、文化繁荣、社会和谐、人民生活富裕的现代化国际城市。

　　2008年，是新一届政府的开局之年，是全面完成“十一五”规划承上启下的重要一年。经济社会发展预期目标是：地区生产总值增长15%；万元GDP综合能耗降低4%，主要污染物排放总量降低3%；固定资产投资增长20%；地方财政一般预算收入增长16.5%；社会消费品零售总额增长15%；城镇居民人均可支配收入、农村居民人均纯收入均增长12%。为实现上述目标，要切实抓好以下工作：

　　1、着力强化国际航运中心的辐射服务功能。落实《大连东北亚国际航运中心发展规划》，以核心港区建设为重点，继续推进航运设施体系、综合运输体系和航运服务体系建设，增强城市辐射能力，发挥在东北振兴和对外开放中的龙头作用。

　　充分发挥保税港区优势，依托口岸、物流、加工功能，大力开展国际贸易、中转、配送等业务。加快电子产品分拨、冷藏物流等物流中心建设，全面提升整体功能。构建以保税港区为核心、以腹地保税物流基地和内陆干港为节点的东北保税物流网络，扩大辐射范围，引领东北振兴。依托东北主要城市的陆路干港，将海铁联运业务向俄罗斯和欧洲延伸，构建亚欧大陆桥便捷通道。

　　加强港口基础设施建设。推进大窑湾和长兴岛核心港区航道、防波堤工程建设。基本完成大窑湾集装箱码头二、三期建设，推进北岸集装箱码头一期、长兴岛港区、旅顺羊头洼港二期等工程建设，全面启动东部港区开发，推进庄河石城港前期工作，开工建设国际邮轮中心等工程。构建黄、渤海港口经济带，发挥港口对区域经济发展的促进作用。

　　构筑畅通的集疏运体系。确保沈大与丹大高速公路连接线竣工，长兴岛疏港高速公路主体完工。推进长兴岛铁路、金连线和铁路中心集装箱场站建设。加快哈大客运专线建设，做好东北东部铁路、新铁路客运站等项目前期工作。

　　加快机场三期和空港物流园建设，进一步增加国际国内航线和航班密度，强化东北亚门户枢纽机场功能。推进新机场前期工作。

　　进一步改善口岸通关环境，完善大通关制度，加强口岸信息化建设，提高口岸服务质量和通关效率。

　　2、进一步优化产业结构。加快产业结构优化升级，着力抓好一批带动能力强、市场潜力大、附加值高的大项目，建设具有国际竞争力的现代产业基地。

　　提高装备制造和石化产业的国际竞争力。依托“两区一带”，形成以骨干企业为龙头、完善的装备制造业和造船业的产业集群。大窑湾临港区域要进一步加快汽车发动机、变速箱等汽车零部件产业发展，争取汽车整车项目的突破。大连湾临海区域要进一步增强聚集效应，形成以海洋工程、核电和风电设备为主的国际一流的重装备制造基地。渤海岸线要加快中远造船、STX造船等船厂的建设速度，积极推进船用柴油机、船用曲轴等配套产业发展，尽快形成中国最大的造船基地。要依托长兴岛临港工业区，形成新的石化产业基地，加快百万吨乙烯项目的前期工作。

　　加快高新技术产业发展。以英特尔项目为突破口，大力发展集成电路及配套产业，形成集人才培养、设计研发、生产加工、封装测试、物流配送为一体，配套企业集聚、国际一流的集成电路产业基地。依托大连的科技人才优势，大力发展以新型能源、节能环保、生物工程为主的高新技术产业，形成新的经济增长点。

　　大力发展现代服务业。努力构建与东北亚重要国际城市相适应的现代服务业体系。以争创第一为目标，加快发展软件和信息服务业，推进旅顺南路软件产业带和大东沟服务外包基地建设，加大软件人才工作力度，进一步优化政策环境，增强软件和服务外包的国际竞争力。促进电子信息产业与媒体、电信等产业结合，拓展主导产业服务功能。加快发展现代物流业，引进更多的国际物流旗舰企业，建设油品、粮食、矿石等资源性商品集散地。加快发展现代金融业，推进星海湾金融商务区建设，吸引优秀金融企业进驻，增强金融业的服务和辐射功能。发展奥运旅游，吸引更多的国内外游客，推进莱茵海岸、世茂嘉年华、金石文化产业基地和老虎滩渔人码头等重大旅游项目建设。进一步提高会展业的专业化、规模化、国际化水平。继续大力发展现代商业，推进电子商务、网上购物等现代商务建设。重点发展研发、安装、售后服务等装备制造服务产业，构建国内知名的生产性服务平台。积极推进文化、设计、策划、新媒体等创意产业发展。

　　3、实施创新驱动战略。以全面提升城市核心竞争力为目标，充分调动企业创新积极性，深入挖掘科技和人才潜力，提高我市优势产业的自主研发能力，抢占自主创新的制高点，使创新成为我市新时期经济发展的强大引擎。

　　建设企业为主体的创新体系。积极引导创新要素向企业集中，支持企业建立市级以上工程中心10家、技术中心23家。围绕重大技术攻关项目，组建以企业为牵头的产业技术联盟，形成产学研相结合的利益共同体。积极引进国内外风险投资基金，大力推进科技型企业在创业板块上市，培育孵化创新型科技企业1500家。加快中科院大连科技创新园建设，形成新的科技创新平台。

　　攻克装备制造业自主研发难关。整合多渠道资金，集中企业、科研院所和高校力量，重点开发一批反映我市装备制造业核心竞争力、具有自主知识产权的新产品。完成3兆瓦风电机组研发并批量生产，建设5000台风电增速器研发生产基地；突破90型船用曲轴技术难关，实现产业化；在高档数控机床研发方面取得新的突破，进一步扩大市场占有份额；全力推进3000米深水半潜式钻井平台和9600千瓦大功率电力机车研制与批量生产。全市装备制造骨干企业要在自主研发上走在全国前列。

　　通过开放式创新抢占技术制高点。紧紧抓住世界产业转移和技术流动的历史机遇，创造良好的人才环境和政策条件，把大连打造成国际技术转移的承接地。积极利用英特尔项目落户大连所形成的技术需求拉动和创新氛围的影响，引进和培养一大批芯片设计、设备制造和相关联IT技术的科技型企业，引进并消化一批世界高端技术。充分利用我市近年来软件外包所形成的人才储备，以高新园区为创新平台，通过政策引导、政府采购、知识产权保护等一系列措施，培育一大批具有自主知识产权的嵌入式软件和应用软件产品，并带动工业设计和动漫技术的自主研发和产业化。

　　4、加快县域经济发展和新农村建设。继续把“三农”工作摆到重中之重的位置，加大支农惠农力度，统筹城乡发展，建立以工促农、以城带乡的长效机制。

　　继续推进县域工业发展。加快“一岛十区”新型工业园区建设，完善基础设施，提高承接能力，推进工业向园区集中。培育壮大装备制造、服装、家具和农产品深加工等优势产业，保持县域经济发展的强劲势头。

　　大力发展现代农业。坚持走中国特色农业现代化道路，创新工作思路，转变发展方式。全面优化农业结构，采取更加有力的措施做强做大水产、畜牧、蔬菜、水果及花卉五大优势产业。支持农业龙头企业、专业合作组织发展，完善农业社会化服务体系。加强动植物疫病防控体系建设，加大农业技术推广力度，促进农业科技进步。

　　扎实推进新农村建设。加强农村基础设施建设，完善农村公路网，实施农村饮水工程。改善农村生态环境，完成植树1亿株，建设八大林业生态重点工程。注重农村新能源建设，新建沼气工程8000个。完成900户生态移民搬迁。大力发展农村旅游业。启动新农村人才振兴计划，加强农村实用人才培养和城乡专业技术人才交流，积极引导农村劳动力转移就业，促进农民持续增收。加大扶持力度，帮助北部山区贫困农民尽快脱贫致富。

　　5、继续深化各项改革。加快重点领域和关键环节的改革步伐，进一步完善社会主义市场经济体制。

　　继续推进国有经济战略性调整。深化国有企业股份制改革，推动国有资本向优势产业和重点企业集中。深化国有企业管理体制和经营机制改革，完善企业自主创新体制。健全国有资产监管体系和行业综合绩效考核评价机制，规范国有资产监管和产权交易，把大连产权交易所建成东北地区产权交易中心，建立国有资本经营预算制度。

　　加快财政、金融体制改革。加大对公共服务领域的投入，增强财政支出的公共性、合理性和均衡性。建立新型投资体制，促进投资主体多元化。建立健全资源有偿使用制度和生态环境补偿机制。加快建立高效安全的现代金融服务体系。深化农村信用社改革，做好新型农村金融机构和农业保险试点工作。加快发展多层次资本市场，扩大直接融资，全力推动企业在境内外上市。

　　加快行政管理体制改革和事业单位分类改革。推进甘井子区、普兰店市的综合配套改革试点工作。探索建立有机统一的大部门体制，精简和规范各类议事协调机构及其办事机构。搞好行政执法类事业单位改革，健全政府职责体系，更加注重履行社会管理和公共服务职能。推进文化事业单位体制改革。

　　继续优化民营经济发展环境。加大政策扶持力度，完善民营经济服务体系，加快建设小企业创业基地、中小企业公共服务平台、特色大厦和特色园区。支持民营中小企业上市融资，建立小企业授信风险补偿奖励机制，鼓励银行为中小企业融资，推动民营中小企业做强做大。

　　6、积极拓展对外开放的广度和深度。进一步扩大开放领域、优化开放结构、提高开放型经济水平，加快城市国际化进程。

　　坚持为我所用的对外开放原则，围绕提升城市核心竞争力，积极引导外商投资向现代产业基地集聚，重点引进高新技术产业、先进制造业、现代服务业和节能环保产业等项目。把我市的产业纳入全球产业链条之中，引导有实力的大企业与跨国公司在基础设施和公共服务领域开展战略合作。加强对外交流与合作，精心组织好亚太城市首脑会议、日韩“大连周”、中欧投资贸易洽谈会和一系列专项招商引资活动，使今年利用外资再上一个新台阶。全年引进投资额1000万美元以上的外资大项目100个以上，优势产业项目注册外资和实际使用外资比重达到80%以上，实际使用外资增长10%。力争再引进一批世界500强企业的地区总部、研发中心、投资性公司和外资金融机构。加快转变外贸增长方式。支持国有大中型企业和民营企业拓展市场、增加出口，扩大高新技术产品、机电产品出口，促进加工贸易转型升级，大力发展以外包为重点的服务贸易，确保外贸进出口总额增长15%。

　　进一步强化开放先导区的示范带动作用。开发区要依托重大产业项目，引进配套企业，促进现代产业集聚和升级，打造具有国际竞争力的临港现代产业基地。保税区要利用政策功能优势，积极引进物流、贸易、临港加工、金融服务等项目，提升辐射水平，争创中日韩自由贸易区先行区。高新园区要以软件产业带建设为重点，大力发展软件和信息服务、工业设计、网络和动漫产业，努力建设世界一流的软件和服务外包基地。坚定不移地实施“五点一线”沿海经济带开发开放战略，长兴岛临港工业区和花园口工业园区要尽快引进一批具有产业牵动性的外资大项目，实现开发开放的重大突破。市内各区要强力推进现代服务业招商引资，围绕提升城市功能，大力发展楼宇经济和总部经济。旅顺口区、长海县要充分利用对外开放的契机，全力打造开放型经济。

　　7、努力提高生态宜居城市建设水平。以城市总体规划修编为契机，加快现代化城市基础设施建设，增强城市的综合服务功能，提高宜居环境水平。

　　进一步加大交通基础设施的投入，切实解决城市交通瓶颈和停车难问题。开工建设海底隧道和扩建香炉礁立交桥等路桥项目，启动滨海路西段、凌川路和市体育中心区域路网建设，改善新老市区连接和西部交通状况。完成城市地铁项目前期工作，争取国家批准并年内开工。完成快轨三号线续建工程，加快推进至旅顺口区、普兰店市延伸线前期工作。开工建设大、小长山岛跨海大桥。

　　加大城市电、水、气等基础设施建设改造力度。加快推进红沿河核电站建设，做好庄河电厂二期前期工作。完成旅顺南路供水工程，开工建设大伙房水库输水入连工程，加快三道沟净水厂改扩建、金州青山水厂二期、登沙河水厂建设和城市供水、供气管网改造。

　　切实强化城市管理。大力推进信息技术在城市管理中的应用，推进城市“一卡通”工程，加快数字化城市建设步伐。巩固城市综合整治成果，加大执法力度，严肃查处违规建设和破坏城市环境等违法行为。

　　优化生态环境。完善夏家河海滨公园，推进前关湿地公园、大黑石海滨公园建设，加快形成城市北部的黄金海岸。建设大连湾森林公园，启动旅顺英歌石森林公园，续建西郊国家森林公园，改造星海公园。人均公共绿地达到11.6平方米，城市绿化覆盖率达到44%。

　　8、强力推进节能减排工作。把节能减排作为促进科学发展的重要环节，健全工作监督和奖惩制度，构建以政府为主导、企业为主体、全社会共同推进的工作格局。

　　着力抓好重点领域的节能。推进百家高耗能企业能源审计，加快用能设施和现有非节能建筑改造，严格实行新建筑节能设计标准，继续推动环境友好型住宅建设。

　　推广应用节能、节水新技术与新产品。继续实施十大节能、节水示范工程，推进水源热泵等节能项目，扩大　　污水处理再利用和雨水利用范围，开发和引进先进海水淡化技术。

　　继续加大减排工作力度。加快东北特钢大连基地、大化集团搬迁改造。彻底淘汰粘土砖、造纸等落后产业。严格控制新增污染物总量，推广清洁和可再生能源。新建和改扩建大连湾、小平岛等污水处理厂，生活污水集中处理率再增加2个百分点，加快“一岛十区”污水处理厂等环境基础设施建设。加大供热并网工作力度，拆除锅炉150台。加强排污总量控制管理，启动国家林业碳汇基金大连专项，实施重点污染源在线监控，实行严格的超标超量排放处罚制度。继续推进农村乡镇生活污水和垃圾综合治理示范工程。

　　9、积极推动社会事业发展。坚持经济社会统筹协调发展，努力办好人民满意的教育、卫生、文化、体育等各项社会事业。

　　深入开展以社会主义荣辱观教育为核心内容的思想道德建设，倡导社会文明新风尚。扎实开展新一轮全国文明城市和文明行业创建活动，完善未成年人思想道德教育体系，深入推进农村思想道德建设。进一步加强军政军民团结和双拥优抚安置工作。

　　促进城乡教育协调、均衡发展。免除城区义务教育学杂费，完成北三市提高普九任务。改造大连八中、二中危旧校舍，在农村偏远地区建设四所寄宿制学校。加强农村远程教育和现代化教育设施管理，推进农村教育现代化。调整中等职业教育公用经费标准，提高职业教育质量。全力支持在连高校发展，围绕建设创新型城市培养更多的创新人才。

　　进一步完善公共卫生服务体系，提高疾控服务和卫生应急能力。加强医疗行业管理，规范服务行为。完善新型农村合作医疗制度，筹资标准提高到120元。加大社区卫生服务体系建设投入，提高综合服务水平。深入开展爱国卫生运动，建设健康城市。提高出生人口素质，推进人口生态平衡。

　　努力繁荣文化事业，积极发展文化产业。营造有利于出精品、出人才的文化环境。加快推进现代博物馆改陈，人民文化俱乐部、人民剧院改造和汉墓博物馆建设，加大对乡镇文化中心和文化站的投入，构建覆盖全社会的公共文化服务体系，满足人民文化需求。精心办好第十七届中国金鸡百花电影节和中日韩文化产业论坛。

　　广泛开展全民健身运动。推进体育中心和新市民健身中心建设，实施国家“农民体育健身工程”和“体育进机关、企业、社区工程”，开展百万学生阳光体育活动。组织好奥运火炬大连接力传递工作，办好大连国际马拉松和国际徒步大会等重大赛事与活动。提高竞技体育水平。

　　全面加强社会管理。完成第七届居委会换届选举工作，加快推进农村社区建设和村委会办公服务设施改造。开展和谐社区、村屯创建活动，大力发展社区服务。健全利益协调和矛盾调处机制，推进“五五”普法工作。加强社会治安综合治理，防范和打击违法犯罪活动，保持社会持续稳定。加大安全投入，强化安全管理和监督，严防重特大安全事故发生。加强市场监管，深入开展虚假违法广告专项整治，严厉打击传销活动。

　　10、切实保障和改善民生。加大对重点民生工程的投入，让人民群众从经济发展成果中得到更多实惠。

　　全面贯彻《就业促进法》，落实积极的就业扶持政策。健全小额担保贷款、税费减免和创业培训的创业扶持体系，大力推动创业就业。建设大连技师学院，积极开展职业培训，加快培养紧缺的高技能人才，推动素质就业。完善就业援助工作机制，开展协约式服务，重点做好零就业家庭等困难群体就业。积极推进国家统筹城乡就业试点工作。搞好高校毕业生就业培训，对困难家庭毕业生实施就业援助。全年实现城镇就业14万人，城镇登记失业率控制在3%以下。

　　完善覆盖城乡的社会保障体系。积极探索建立农村居民、城镇居民和农民工养老保险制度。今后连续三年提高企业退休人员养老金标准。全面建立城镇居民医疗保险制度，将大学生纳入医疗保障体系，进一步提高医疗保险参保人员待遇。认真贯彻《劳动合同法》，实施劳动关系和谐工程，建立劳动用工备案制度，实行劳动保障监察网络化管理，保障劳动者合法权益。

　　健全城乡社会救助体系。提高城乡低保标准，落实城市低收入家庭专项救助制度。将困难居民全部纳入城乡医疗救助体系，帮助农村困难居民改造危房2000户。完善市县两级老年活动基础设施和服务体系，出台城市特困老人居家养老服务补贴和民营养老机构新增床位补贴政策。新建市福利院养老特护院和农村区域性中心敬老院。加大老年人、残疾人、孤残儿童等特殊群体的社会福利供给，构建覆盖城乡的社会福利服务体系。完善城乡灾害应急救助救援机制和救助管理社会化服务网络。充分发挥慈善组织的积极作用，多渠道吸引社会资金投向公共服务领域。

　　把“居者有其屋”作为民生工作的重点，实现住房保障工作新突破。继续加大住房保障的投入，增加投资2000万元，将市内四区低收入住房困难家庭全部纳入廉租住房保障范围，彻底解决困难群体住房问题。各区市县住房困难的低保家庭必须做到应保尽保。有条件的区市县也要逐步将低收入住房困难家庭纳入保障范围。建设经济适用房和廉租房80万平方米。启动新居工程，对主城区的危旧住宅和弃管房进行改造，切实改善居民的居住条件。加强对房地产市场的调控和引导，增加中小套型普通商品房供应，打击房地产市场投机行为。

　　推进“食品安全城市”建设。深入实施“食品放心工程”，强化检验检测体系建设，完善食品安全监管机制，确保食品安全。采取多种措施，抓好菜篮子工程，稳定粮油及副食品供应，努力控制市场价格。

　　11、深入抓好政府自身建设和管理创新。巩固和扩大“行政效能年”建设成果，深化行政体制改革，提高公共服务能力。

　　大力推行政务公开。认真贯彻《中华人民共和国政府信息公开条例》，扎实推进政府信息公开、行政权力运行公开、公共企事业单位办事公开和基层公开，切实保障人民群众的知情权、表达权、诉求权和监督权。

　　进一步深化行政审批制度改革，全面清理非许可审批事项，落实年检制度改革措施，优化办事流程，切实减轻企业和基层负担。

　　加强市、县两级行政服务中心和四级公共服务体系建设，强化功能，完善机制，继续推广“一站式”和“全程代理制”服务，努力为企业、基层和人民群众提供办事方便。

　　认真落实行政执法责任制，切实推进依法行政。加强电子政务建设，推广网上行政审批。加快应急预案体系和信息平台建设，切实增强应急管理能力。建立运行通畅、监督有力的行政投诉工作网络和机制，加强行政监察和审计监督，规范和约束政府行政行为。全面推行ISO9000质量管理体系认证工作，实现流程再造和管理创新。强化教育培训管理，全面提高公务员素质。

　　自觉接受人大的法律监督、工作监督和政协的民主监督。继续坚持重大事项向人大报告、向政协通报制度，认真办理人大常委会的监督意见、人大代表建议和政协提案，启动建议提案网上管理系统，提高办理工作效率。

　　各位代表，建设一个高效廉洁、务实为民的政府，是600万大连人民的期望和重托。各级政府和广大公务员要时刻牢记为人民服务的宗旨，坚持以人为本、执政为民，常修为政之德，常思贪欲之害，常怀律己之心，恪尽职守，廉洁从政，勤奋工作，为全市人民创造更多的福祉，为大连的发展做出更大的贡献。

　　各位代表，大连又站在了新的历史起点，我们正面对光辉灿烂的美好未来。让我们紧密地团结在以胡锦涛同志为总书记的党中央周围，高举中国特色社会主义伟大旗帜，全面贯彻落实科学发展观，推进经济社会又好又快发展，加快构建和谐大连，为率先实现老工业基地全面振兴，把大连建设成为东北亚重要的国际城市而努力奋斗！
